# Giant Asymptomatic Intermuscular Lipoma of Right Thigh: Case Report and Review of Literature

**DOI:** 10.1002/ccr3.72478

**Published:** 2026-04-06

**Authors:** Letho Letho, Nishal Chhetri

**Affiliations:** ^1^ Department of Orthopedics Jigme Dorji Wangchuck National Referral Hospital Thimphu Bhutan; ^2^ Department of Pathology and Laboratory Services Jigme Dorji Wangchuck National Referral Hospital Thimphu Bhutan

**Keywords:** asymptomatic, giant lipoma, intermuscular, surgical resection

## Abstract

Though lipomas are the most common benign soft tissue tumors, giant lipomas are rarely reported in the literature. Giant lipomas should be properly evaluated for any evidence of malignant transformation such as symptomatic, progressive growth in size, heterogeneity, hypervascularity, and irregular septation. Surgical resection should be considered in asymptomatic giant lipomas due to the risk of malignant transformation. Lipomas are the most common benign soft tissue tumors of adipose tissue and are typically small, slow‐growing, and asymptomatic. Giant lipomas, defined as lesions measuring ≥ 10 cm in one dimension or weighing ≥ 1 kg, are rare and may mimic malignancy due to their size and deep location. We report a case of a 58‐year‐old woman who presented with a progressively enlarging, asymptomatic mass over the right thigh for 7 years. Clinical examination revealed a firm mass measuring approximately 20 × 8 cm. Magnetic resonance imaging demonstrated a well‐defined, lobulated intermuscular lipomatous lesion measuring 24 × 8.5 × 7 cm involving the vastus medialis muscle, without features suggestive of malignant transformation or neurovascular encasement. Given the progressive enlargement and giant size of the tumor with local mass effect, marginal en‐bloc resection was performed through an anteromedial approach. Histopathological examination confirmed a benign encapsulated lipoma composed of mature adipocytes without atypia or malignancy. The postoperative course was uneventful, and no recurrence was noted at the 6‐month follow‐up. Although most lipomas are managed conservatively, giant lipomas warrant surgical excision for definitive diagnosis, symptom prevention, and exclusion of malignancy. A careful surgical planning, meticulous surgical resection, and close postoperative follow‐up can ensure excellent functional outcomes and prevent surgical complications.

## Key Clinical Message

Asymptomatic giant lipomas with local mass effect are better managed with surgical resection for cosmetic improvement and to rule out malignancy.

## 1 Introduction

Lipomas are the most common benign soft tissue tumors of adipose tissue, and their development is multifactorial, involving genetic, molecular, and local soft tissue factors [1]. Although the exact pathogenesis is not fully understood, several mechanisms have been proposed [2]. The most widely accepted mechanism involves chromosomal abnormalities affecting adipocyte precursor cells. Many solitary lipomas demonstrate rearrangements involving chromosome 12, particularly the 12q13–15 region. This region contains the HMGA2 (High Mobility Group AT‐hook 2) gene, which plays a role in cell growth and differentiation [3]. Other theories include abnormal adipocyte differentiation, trauma‐induced theory, and endocrine and metabolic influences [4, 5, 6, 7, 8]. Obesity is one of the factors associated with lipoma [9, 10]. It is more common in females and most prevalent at 40–60 years of age [11, 12]. It is commonly seen in the trunk and upper extremities. Usually, it is soft in consistency and small; however, it can become firm and can grow extremely large. Most of the lipomas are asymptomatic and benign. However, in a few cases, giant lipomas can cause symptoms due to compression of adjacent soft tissues, such as nerves and blood vessels. Giant lipomas are defined by measuring at least 10 cm in one dimension and weighing a minimum of 1 kg [13]. The incidence of giant lipomas is very rare, and it accounts for only 1% of all lipomas. Till now, only 10 cases of giant lipomas are reported in the literature [14].

Lipomas are usually diagnosed clinically and simple radiological investigations such as ultrasonography. More advanced imaging modalities like CT and magnetic resonance imaging (MRI) are indicated only when there is clinical suspicion of malignancy. Asymptomatic lipomas are generally treated conservatively with regular clinical observations for any evidence of transformation to malignancy. Giant lipomas are better removed surgically for confirmation of disease and to rule out malignancy. The objective of reporting this case is to highlight the importance of surgical management of progressively growing giant lipomas even if the patient is asymptomatic, how proper evaluation of the patient's clinical information and radiological imaging and meticulous surgical technique can prevent anticipated complications and achieve good clinical outcome.

The unique features of this case lie in several important aspects. Despite measuring 24 × 5 × 7 cm, the tumor remained completely asymptomatic, which is unusual given that most giant lipomas typically present with local pressure effects such as pain, restriction of movement, and occasionally neurovascular compromise. Deep intermuscular lipomas are considerably less common than superficial subcutaneous variants, and in this case, the involvement of nearly the entire length of the vastus medialis muscle renders it anatomically remarkable. Furthermore, the successful complete excision of the tumor without preoperative biopsy confirmation reflects sound clinical judgment and careful surgical decision‐making, particularly within a resource‐limited setting such as ours.

## 2 Case Presentation

A 58‐year‐old woman presented to the orthopedic outpatient department with a history of a progressively enlarging swelling over her right thigh for the past 7 years. She initially noticed a small, painless lump that was insidious in onset and did not interfere with her daily activities. As it was asymptomatic at the beginning, she did not seek medical attention.

Over the years, the swelling gradually increased in size without any history of trauma, fever, weight loss, or constitutional symptoms. She denied pain, redness, or discharge from the swelling. There was no history suggestive of rapid recent enlargement. However, with progressive growth, she began experiencing discomfort due to the bulk of the mass, particularly while walking long distances and wearing fitted clothing.

There were no associated neurological symptoms such as numbness, tingling, or weakness in the affected limb, and no features suggestive of vascular compromise. She had no prior history of similar swellings elsewhere in the body and no known family history of soft tissue tumors. She is a farmer and denied smoking tobacco and drinking alcohol.

On general physical examination, the patient was noted to have class II obesity, with a body mass index (BMI) of 36 kg/m2. Local examination of the right thigh revealed a visibly enlarged thigh compared to the contralateral side. The overlying skin appeared smooth and shiny, with no evidence of ulceration, discoloration, or dilated veins. On palpation, there was no local rise in temperature. A diffuse, nontender mass was palpable over the anteromedial aspect of the right thigh. The swelling measured approximately 20 × 8 cm and extended from the medial aspect of the knee joint proximally toward the groin. The mass was firm in consistency, mobile in the horizontal plane, but relatively fixed in the vertical plane, suggesting deep intermuscular involvement. Distal neurovascular examination was unremarkable. The popliteal artery pulse was palpable, and motor as well as sensory functions of the right lower limb were intact. The range of motion of both the right hip and knee joints was within normal.

## 3 Investigation

Baseline laboratory investigations revealed a total white blood cell count of 7.2 × 10^3^/μL (reference range: 4.21–10.29 × 10^3^/μL), erythrocyte sedimentation rate (ESR) of 47 mm/h (0–15 mm/h), and C‐reactive protein (CRP) level of 1.6 mg/L (0.00–6 mg/L). Her lipid profile showed elevated total cholesterol of 241 mg/dL (150–220 mg/dL) and low‐density lipoprotein (LDL) cholesterol of 162 mg/dL (0–130 mg/dL), with high‐density lipoprotein (HDL) cholesterol of 44 mg/dL (42–88 mg/dL).

**FIGURE 1:**
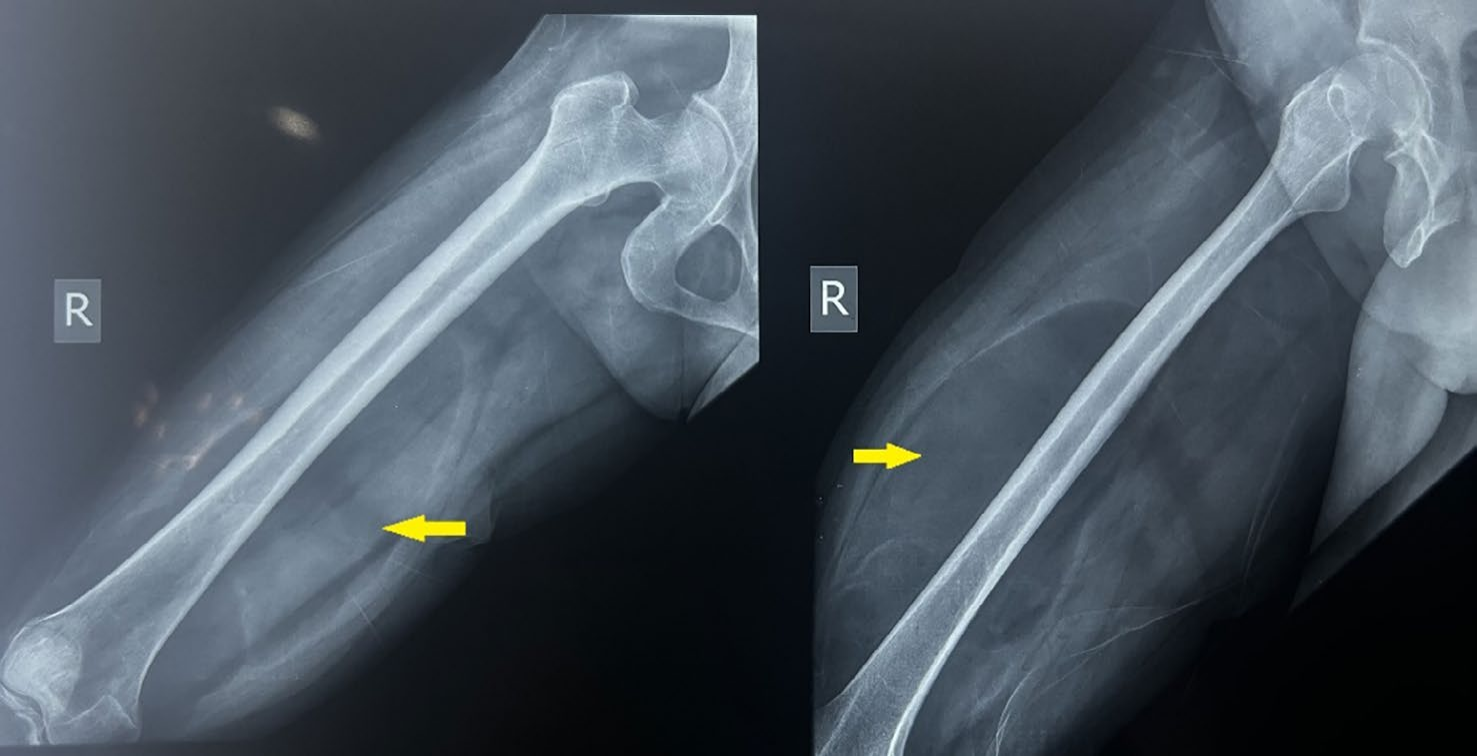
Plain radiographs of the right thigh in anteroposterior and lateral views demonstrate a well‐defined soft tissue radiolucent area along the medial to anteromedial aspect of the thigh (as indicated by yellow arrows). The lesion appears as an oval, homogeneous region of decreased soft tissue density consistent with fat attenuation.

Plain radiographs of the right thigh in anteroposterior and lateral views demonstrated a well‐circumscribed, oval‐shaped radiolucent lesion consistent with a fat‐density mass located over the anterolateral aspect of the right thigh, extending posteriorly to the distal two‐thirds of the femur (Figure 1). There was no evidence of cortical erosion or periosteal reaction.

**FIGURE 2:**
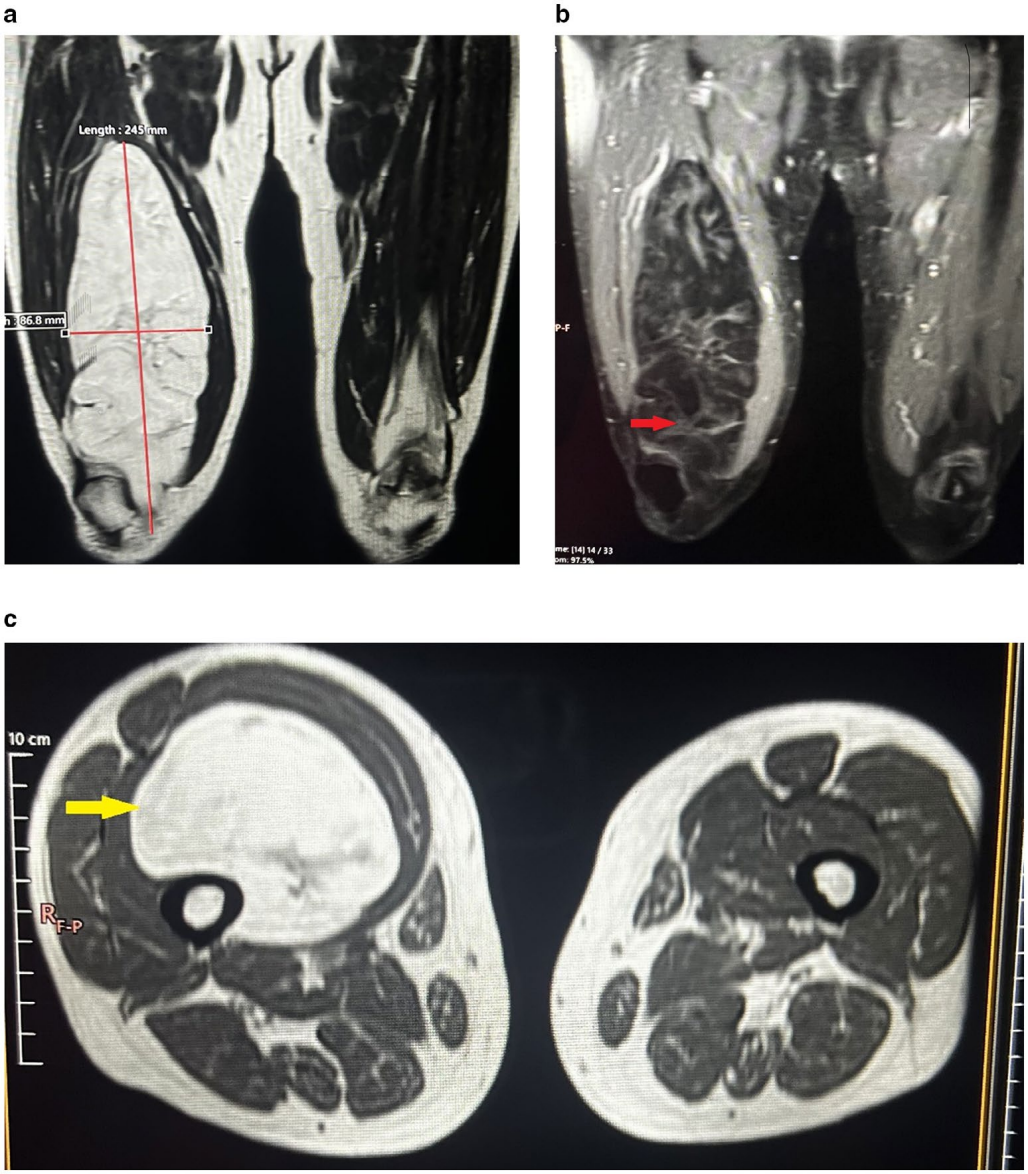
(a) Magnetic resonance imaging of the right thigh (coronal section) demonstrates a large, well‐defined, lobulated intramuscular mass located along the medial aspect of the thigh. The lesion measures approximately 24.5 cm in craniocaudal length and 8.6 cm in transverse diameter. The mass appears homogeneously hyperintense on T1‐weighted imaging, with signal characteristics identical to subcutaneous fat, consistent with a lipomatous lesion. (b) MRI right thigh coronal view with contrast showing no contrast enhancement with irregular septation of mass (red arrow). (c) MRI right thigh axial view T2‐weighted image showing well capsulated soft tissue mass at right thigh (yellow arrow).

MRI of the right thigh revealed a large, lobulated, well‐defined lesion measuring approximately 24 × 8.5 × 7 cm. The mass appeared hyperintense on both T1‐ and T2‐weighted sequences and involved nearly the entire length of the vastus medialis muscle, consistent with a large intermuscular lipomatous lesion (Figure 2a,c). No significant enhancement was observed following intravenous gadolinium administration (Figure 2b). There was no evidence of invasion or encasement of adjacent neurovascular structures.

## 4 Management

In view of the progressively enlarging mass and increasing functional discomfort due to local mass effect, the patient was planned for marginal excision of the tumor for symptomatic relief, cosmetic improvement, and definitive histopathological diagnosis. The procedure was performed under spinal anesthesia. The patient was positioned supine with the hip slightly abducted and externally rotated to optimize exposure of the anteromedial thigh. As a tourniquet was not utilized, measures to minimize intraoperative blood loss included local infiltration with 2% lignocaine containing adrenaline, meticulous layer‐by‐layer dissection, and careful cauterization of bleeding points.

**FIGURE 3:**
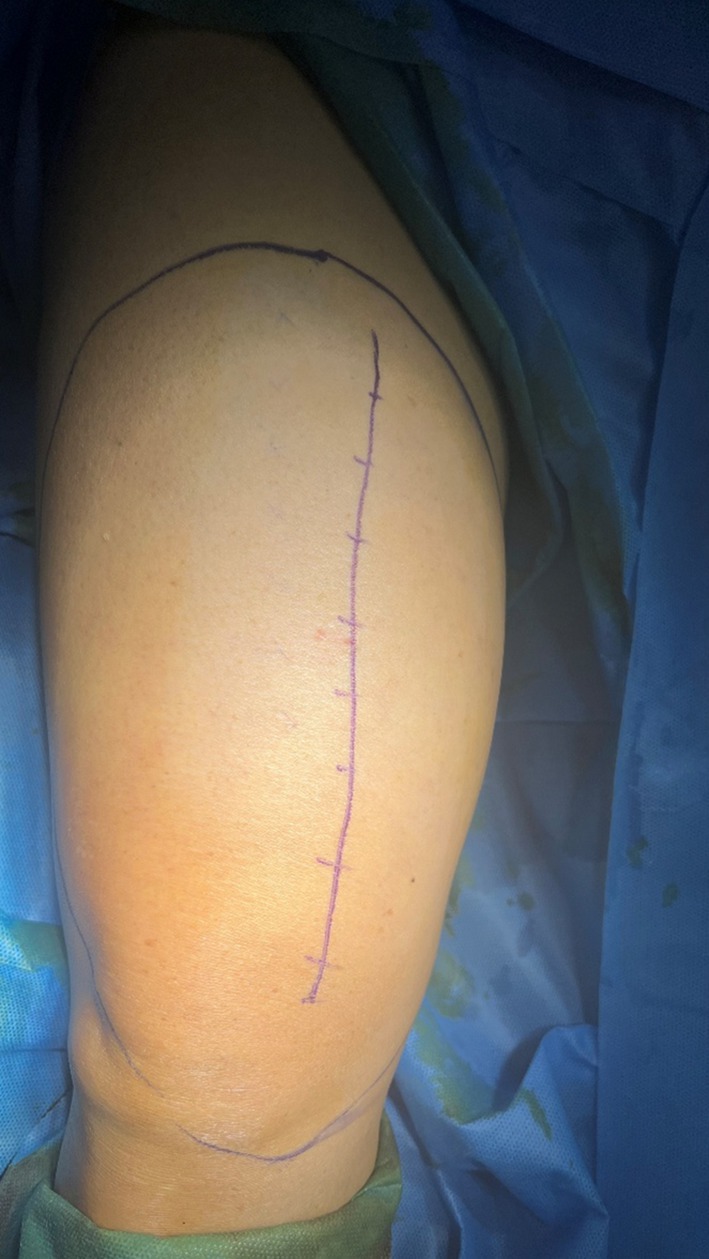
Intraoperative image showing the extent of the tumor margins and line of surgical incision.

A longitudinal incision was made over the anteromedial aspect of the thigh directly overlying the tumor. Following division of the deep fascia, the lesion was approached through the intermuscular plane between the rectus femoris and vastus medialis muscles (Figure 3). Given the proximity of the femoral neurovascular bundle medially, exploration was performed under direct visualization. Dissection was carefully confined within the tumor pseudocapsule. Blunt dissection using a surgical mop was employed to gently separate the mass from the surrounding muscle fibers, and excessive traction, particularly along the medial aspect, was strictly avoided to prevent neurovascular injury.

**FIGURE 4:**
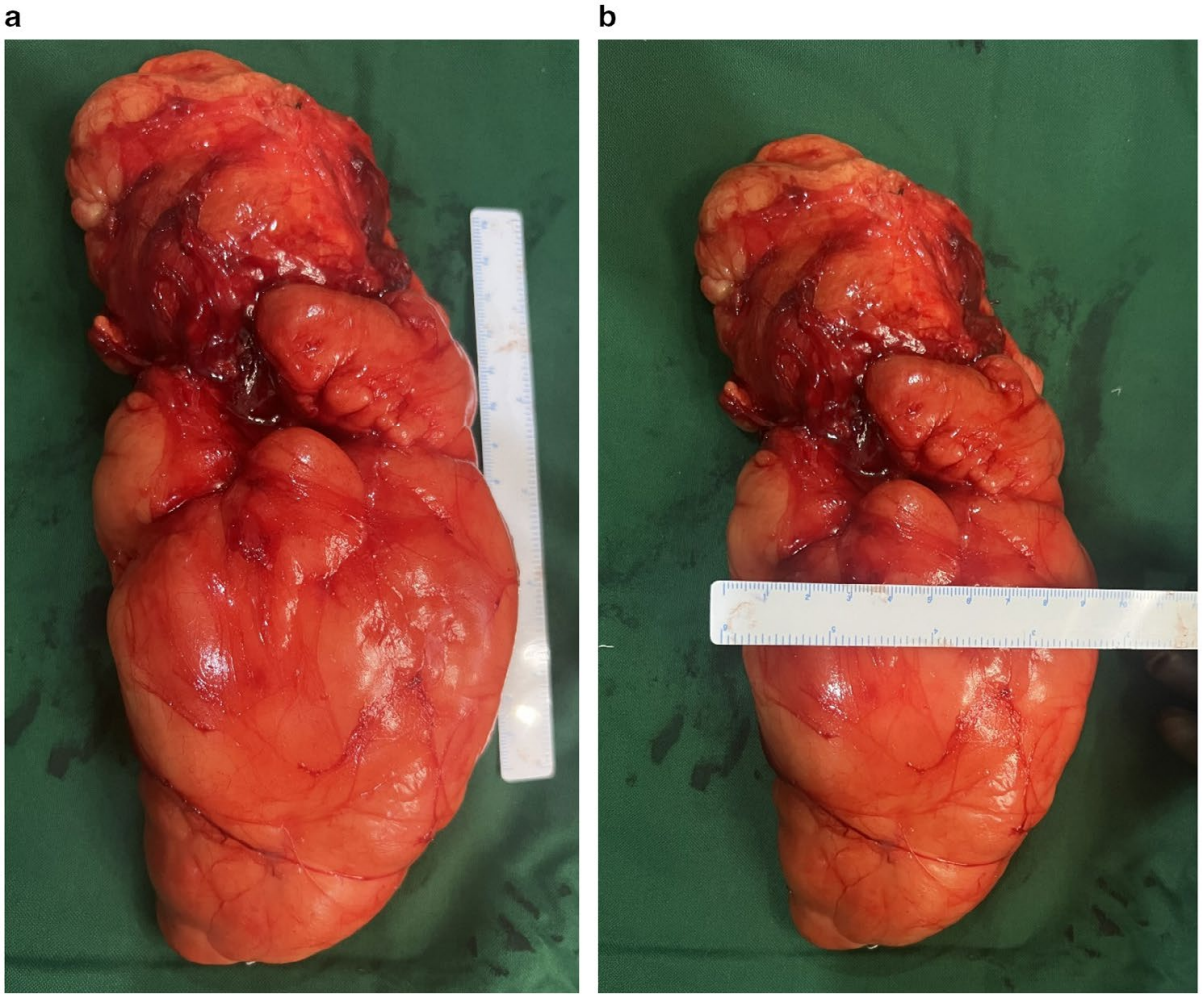
(a) Large well‐circumscribed, multilobulated soft tissue mass with a smooth surface and encapsulated and covered by a thin, glistening fibrous capsule measuring 24 cm. The external surface shows a yellow to yellow to orange coloration, consistent with adipose tissue. (b) The width of the tumor is approximately 8 cm.

Intraoperatively, a well‐encapsulated mass was identified without evidence of invasion or encasement of adjacent muscles, neurovascular structures, or bone (Figure 4a,b). The tumor was excised en bloc and sent for histopathological examination. The surgical field was thoroughly irrigated, hemostasis secured, and a closed negative‐pressure drain was placed prior to routine layered wound closure. Postoperatively, distal neurovascular status remained intact. The patient was mobilized on the same day and discharged on postoperative day 2. The wound healed uneventfully, and sutures were removed on postoperative day 10.

**FIGURE 5:**
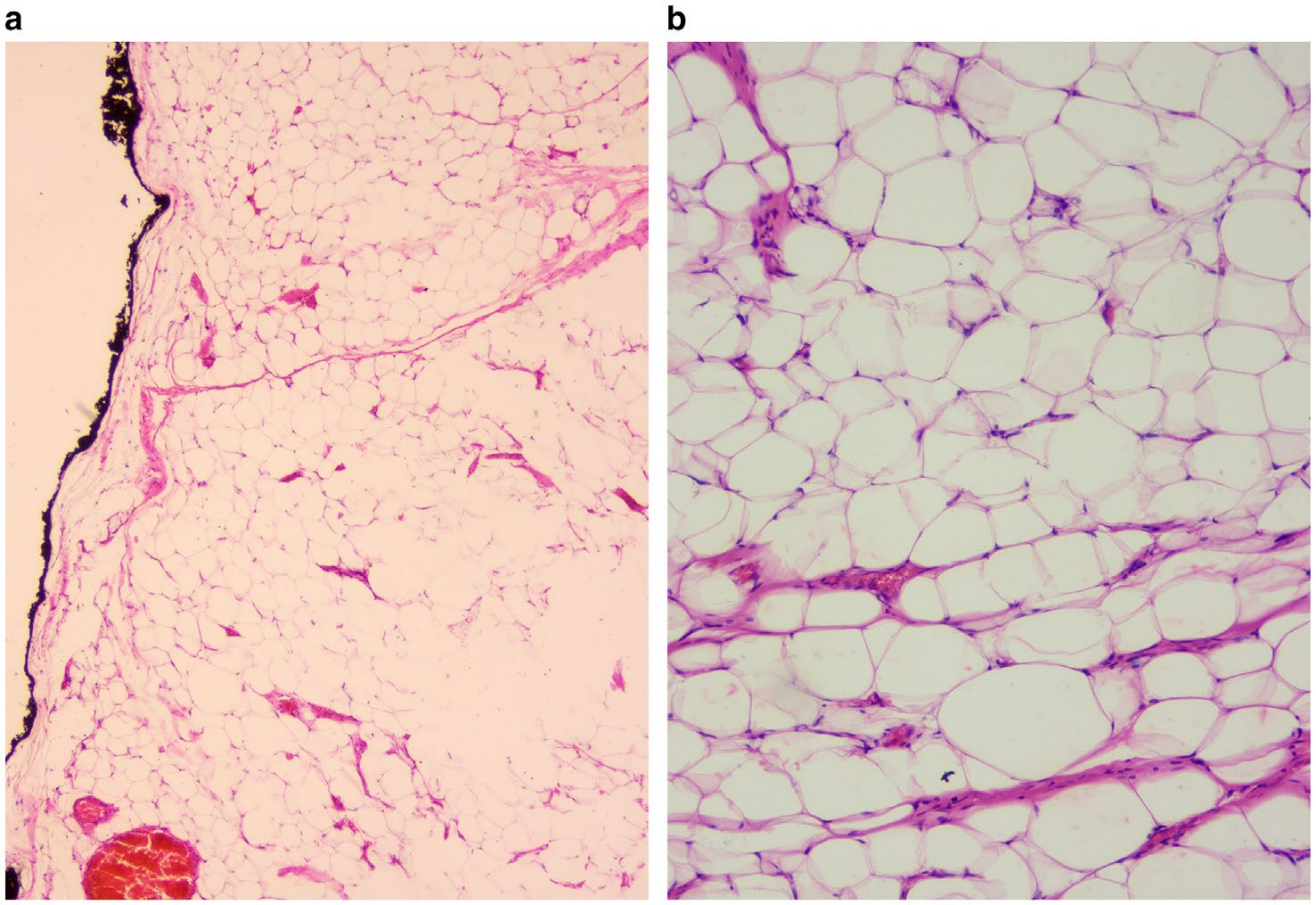
(a) Microscopic images of the Lipoma (H&E stain): A well‐circumscribed lipoma with thin fibrous capsule (inked) with homogeneous proliferation of mature adipocytes and paucicellular fibrous septa (40×). (b) Homogenous population of mature adipocytes, with variably sized paucicellular fibrous septa, but no cytological atypia seen (400×).

Gross examination of the specimen revealed a well‐circumscribed mass with a uniform, glistening yellow to pale‐tan cut surface. Microscopic evaluation demonstrated a well‐encapsulated lesion composed of mature adipocytes with eccentrically placed benign nuclei, without cellular atypia, lipoblasts, or features of malignancy, consistent with a benign lipoma (Figure 5a,b). The immunohistochemical analysis of the tumor to confirm its benign nature could not be performed due to a lack of required facilities at our center.

## 5 Outcome and Follow Up

Post operatively she was discharged 2 days following the surgery. She was followed up after 1, 3, and 6 months at our Orthopedic outpatient department. Her wound healed and she reported no surgical related complications. At her last follow up at 1 year, she had no pain or recurrence of mass.

## 6 Discussion

Most of the asymptomatic lipomas can be treated coservatively [2, 6, 7, 8, 9, 15]. The general indications for surgical excision are cosmetic reasons, histopathological confirmation of disease, symptomatic lipomas, when the size is more than 5 cm or when it is rapidly progressing in size and when there is radiological evidence of malignant transformation [12].

Johnsons et al. recommended that any soft tissue tumor, if the size is more than 5 cm, should be considered malignant until proven otherwise [16]. The characteristics of malignant transformations are large size, irregularly thickened septa, heterogeneity, increased vascularity, and low‐fat content [13]. In the presence of such features, a preoperative FNAC or core biopsy is warranted before undergoing surgical resection. FNAC is relatively cost‐effective, with good sensitivity of 96% and specificity of 98% for the diagnosis of lipoma [17]. Preoperative tissue diagnosis not only aids in confirming the benign nature of the lesion but also facilitates appropriate surgical planning, including the extent of resection and the need for oncological margins. In cases where imaging findings are equivocal or suggest deep intermuscular involvement, histopathological confirmation before definitive surgery becomes particularly important to prevent inadequate excision or the need for reoperation [5]. However, in our case, no FNAC or biopsy was done since there were no other features of malignant transformation such as heterogeneity, hypervascularity, and clinical symptoms in the MRI. In addition, the patient comes from a faraway remote place, and it takes substantial time to get the biopsy report. From our experience, the majority of patients do not turn up for follow‐up with the biopsy report, especially when the conditions are asymptomatic. Therefore, we decided to go ahead with one‐stage tumor resection after thorough discussion with the patient.

Traditionally, symptomatic lipomas are usually excised surgically; recently, other minimally invasive procedures such as liposuction and suction‐assisted lipectomy are proposed. However, these procedures have few drawbacks especially in resection of giant lipomas such as limited visualization of tumor, intraoperative fragmentation of tumor and inadequate excision leading to more risk of recurrence [18, 19].

The common surgical complications that should be anticipated during resection of such giant tumor are excessive bleeding, injury to important neurovascular structures, and surgical site infection. Administration of prophylactic antibiotics, proper preoperative planning, review of imaging reports of the extent of the tumor and status of surrounding neurovascular structure, meticulous surgical dissection under direct visualization, blunt dissection, and dissection confined to the tumor pseudocapsule are some of the key strategies to mitigate such complications.

The plausible explanation for the rapid increase in tumor size could be attributable to a few factors such as Grade II obesity; she experienced significant weight gain over the last 5 years after she developed osteoarthritis of her knees, which restricted her activities and mobility. The other contributing factors could be the Bhutanese diet, which consists mainly of a high intake of carbohydrates, as rice is the staple food in our culture.

Since the tumor was found to be well capsulated and histopathology did not reveal any malignant transformation, referral to the oncologist was not warranted.

## 7 Limitations

Some of the limitations of this case report are the lack of preoperative tissue diagnosis, immunohistochemical analysis, and relatively short follow‐up period to determine the recurrence.

## 8 Conclusion

We report a first case of giant asymptomatic lipoma of thigh from Bhutan. Though asymptomatic, a surgical resection and biopsy should be considered for giant lipomas due to the associated risk of malignant transformation. Proper preoperative planning and meticulous surgical resection can prevent complications and improve outcomes.

## Author Contributions

**Letho Letho:** conceptualization, data curation, formal analysis, investigation, methodology, project administration, writing – review and editing. **Nishal Chhetri:** writing – review and editing.

## Funding

The authors have nothing to report.

## Ethics Statement

Ethics approval was not required for case report by the Institutional Review Board of KGUMSB.

## Consent

Written informed consent was obtained from the patient for publication of this case report and the accompanying clinical images. All procedures performed were in accordance with the ethics standards of the institutional guidelines and the principles of the Declaration of Helsinki.

## Conflicts of Interest

The authors declare no conflicts of interest.

## Data Availability

The authors have nothing to report.
